# Adaptation of the human auditory cortex to changing background noise

**DOI:** 10.1038/s41467-019-10611-4

**Published:** 2019-06-07

**Authors:** Bahar Khalighinejad, Jose L. Herrero, Ashesh D. Mehta, Nima Mesgarani

**Affiliations:** 1 Mortimer B. Zuckerman Mind Brain Behavior Institute, Columbia University, New York, NY 10027 USA; 2 Department of Electrical Engineering, Columbia University, New York, NY 10027 USA; 3 Hofstra Northwell School of Medicine, Manhasset, NY 11549 USA; 4 The Feinstein Institute for Medical Research, Manhasset, NY 11030 USA

**Keywords:** Auditory system, Cortex

## Abstract

Speech communication in real-world environments requires adaptation to changing acoustic conditions. How the human auditory cortex adapts as a new noise source appears in or disappears from the acoustic scene remain unclear. Here, we directly measured neural activity in the auditory cortex of six human subjects as they listened to speech with abruptly changing background noises. We report rapid and selective suppression of acoustic features of noise in the neural responses. This suppression results in enhanced representation and perception of speech acoustic features. The degree of adaptation to different background noises varies across neural sites and is predictable from the tuning properties and speech specificity of the sites. Moreover, adaptation to background noise is unaffected by the attentional focus of the listener. The convergence of these neural and perceptual effects reveals the intrinsic dynamic mechanisms that enable a listener to filter out irrelevant sound sources in a changing acoustic scene.

## Abstract (web summary)

How does the auditory system allow for accurate speech perception against changes in background noise? Here, using neural activity in the auditory cortex as people listen to speech, the authors provide evidence that background noise is selectively suppressed to enhance representations of speech.

## Introduction

Speech communication under real-world conditions requires a listener’s auditory system to continuously monitor the incoming sound, and tease apart the acoustic features of speech from the background noise^1^. This process results in an internal representation of the speech signal that enables robust speech comprehension unaffected by the changes in the acoustic background^2^.

Studies of the representational properties of vocalization sounds have confirmed the existence of a noise-invariant representation in animal auditory cortex. Specifically, it has been shown that the auditory cortical responses in animals selectively encode the vocalization features over the noise features^3–7^. A noise-invariant representation of speech in the human auditory cortex has also been shown^8,9^, but the encoding properties of speech in noise in humans are less clear due to the limited spatiotemporal resolution of noninvasive neuroimaging methods. Previous studies of the neural representation of speech or vocalizations-in-noise have used constant background noises^3–9^. As a consequence, their findings only show the aftereffects of adaptation and the properties of the neural representation once the noise has been removed. Therefore, it remains unclear how, when, and where adaptation unfolds from moment to moment as a new background noise suddenly appears in or disappears from the acoustic scene. For this reason, many important questions regarding the dynamic properties of adaptation to noisy speech in the human auditory cortex remain unanswered, such as (I) how the invariant representation of vocalizations emerges over the time course of adaptation, (II) how the neural representation and perception of phonetic features change over the time course of adaptation, and (III) how cortical areas with different response properties adapt when transitioning to a new background condition. Answering these questions are crucial for creating a complete dynamic model of speech processing in the human auditory cortex.

Here, we combine invasive electrophysiology and behavioral experiments to shed light on the dynamic mechanisms of speech-in-noise processing in the human auditory cortex. We recorded from high-resolution depth and surface electrodes implanted in the auditory cortex of neurosurgical patients. Using an experimental design in which the background noise randomly changes between four different conditions, we report rapid suppression of noise features in the cortical representation of acoustic scene, resulting in enhanced neural representation and perception of phonetic features in noise.

## Results

### Neural adaptation to changing background condition

**Fig. 1:**
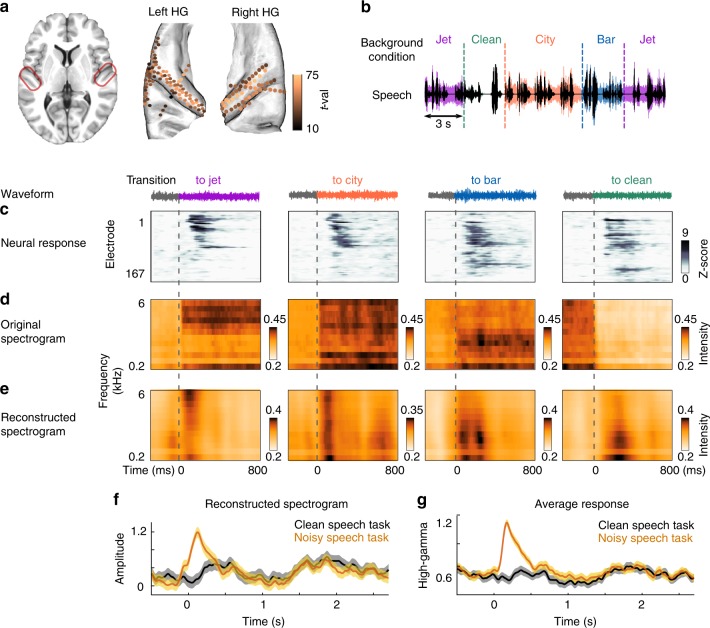
Neural adaptation in transition to a new background condition. **a** Electrodes with significant response to speech (t-val > 10, *t*-test speech versus silence), located in Heschl’s gyrus, the superior temporal gyrus, the transverse temporale sulcus and the planum temporale, shown on a BCI-DNI brain atlas. **b** Experiment design. Clean speech is shown with black; colors show the different background conditions, which change randomly between bar, city, jet, and clean (no noise) every 3 or 6s. **c** The average normalized neural responses aligned by the time of transition (dashed lines) to each background condition (columns). Electrodes are sorted based on the similarity of their adaptation strength using Unweighted Pair Group Method with Arithmetic Mean (UPGMA) algorithm. **d**, **e** Average original spectrograms and reconstructed spectrograms from the neural responses over all transitions to each of the four background conditions. The spectrograms are aligned by the time of transitions. **f** The reconstructed spectrograms averaged over the frequency dimension are shown for clean speech task and noisy speech task. The reconstruction from noisy speech is the average of all three noisy conditions. Shaded error bars indicate standard error. **g** Direct comparison of neural responses to noisy speech task and clean speech task normalized based on the responses in the silence interval. Shaded error bars indicate standard error

We recorded electrocorticography data from six human subjects implanted with high-density subdural grid (EcoG) and depth (stereotactic EEG) electrodes as a part of their clinical evaluation for epilepsy surgery. One subject had both grid and depth electrodes, four subjects had bilateral depth electrodes, and one subject had only grid electrodes (Fig. 1a). Subjects listened to 20 min of continuous speech by four different speakers (two male speakers and two female speakers). The background condition changed randomly every 3 or 6 s between clean (no background noise), jet, city, and bar noises and was added to the speech at a 6 dB signal-to-noise ratio (Fig. 1b). These three types of common background noise were chosen because they represent a diversity of spectral and temporal acoustic characteristics (Supplementary Fig. 1), as is evident from their average acoustic spectrograms shown in Fig. 1d. For example, the jet noise has high frequency and high temporal modulation power, the city noise has uniformly distributed power over frequencies, and the bar noise has mostly low-frequency power. In total, there were 294 transitions between background conditions, distributed evenly among the 4 conditions. The background noise segments were not identical and were randomly taken from a minute-long recording. To ensure that the subjects were engaged in the task, we paused the audio at random intervals and asked the subjects to report the last sentence of the story before the pause. All subjects were attentive and could correctly repeat the speech utterances. All subjects were fluent speakers of American English and were left-hemisphere language dominant (as determined with Wada test).

We extracted the envelope of the high-gamma band (75–150 Hz), which has been shown to reflect the average firing of nearby neurons^10,11^. For all analyses, the electrodes were selected based on a significant response to speech compared with silence (*t*-test, false discovery rate [FDR] corrected, *p* < 0.01). This criterion resulted in 167 electrodes in perisylvian regions, including Heschl’s gyrus (57 electrodes), the transverse temporal sulcus (12 electrodes), the planum temporale (26 electrodes), and the superior temporal gyrus (STG, 39 electrodes), from both brain hemispheres (97 left, 70 right) (Fig. 1a, Supplementary Fig. 2).

To study how the neural responses to speech are affected when the background condition changes, we aligned the responses to the time of the background change and averaged over all transitions to the same background condition. The average response in Fig. 1c shows a short-term transient peak, which occurs immediately after the background changes (average duration = 670 ms, *t*-test, FDR corrected, *p* < 0.01, Supplementary Fig. 3). This short-term response appears in all four conditions, even in the transition to the clean condition (e.g., from speech with jet noise to clean speech). Figure 1c also illustrates that the selectivity and magnitude of this adaptive response to different background conditions varies across neural sites.

### Adaptation suppresses the representation of noise features

To study what features of the acoustic scene are encoded in the responses over the time course of adaptation, we used the method of stimulus reconstruction^12,13^. Reconstruction methods typically rely on learning the linear mapping that relates evoked neural population responses to a time-frequency (spectrogram) representation of sound. This method enables a direct comparison between original and reconstructed spectrograms, making it possible to analyze what spectrotemporal features are encoded at the neural population level. We first trained the reconstruction model on responses to clean speech without the added background noise for each subject separately and used it to reconstruct the spectrograms from the neural responses to speech with added background noises. The reconstructed spectrograms were then averaged across all subjects. We aligned the original and reconstructed spectrograms to the time of the background changes and calculated averages over all trials that shared the same new condition. Comparison of the average original (Fig. 1d) and reconstructed (Fig. 1e) spectrograms shows that immediately after a transition, the neural responses encode the acoustic features of the background noise, which can be seen from the similarity of the reconstructed and original spectrograms after a transition (e.g., the high-frequency energy in jet noise or the low-frequency energy in bar noise). The acoustic features of noises in the reconstructed spectrograms, however, fade away quickly when the adaptation is over, resulting in a noise-invariant representation of speech sounds. This noise-invariant representation is better illustrated in Fig. 1f which shows the temporal shape of reconstructed spectrograms by averaging over their frequency dimension. For comparison, we also presented the same speech materials to the subjects but without any added background noises (clean speech task). The similarity of average reconstructions from the responses in noisy speech task and clean speech task after the adaptation interval is shown in Fig. 1f. Additionally, we also directly compared the neural responses in the noisy and clean speech task and observed the same initial transient divergence of responses after transitioning to a new noise which then converged to the neural response to the clean speech task after the adaptation interval (Fig. 1g).

**Fig. 2:**
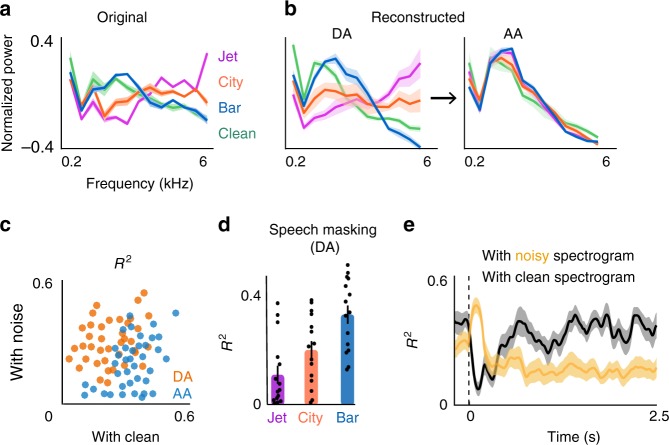
Suppression of noise features after transitioning to a new background condition. **a** Average frequency profile of the four background conditions calculated from the original spectrograms. **b** Average frequency profile of the background conditions calculated from reconstructed spectrograms during adaptation (DA, 0–0.4s after transition) and after adaptation (AA, 2.0–2.4s after transition) (20% cross-validation for each condition, *n* = 5). **c** ${{R}}^2$ between reconstructed spectrograms and the original clean (x-axis) and original noisy spectrogram (y-axis) for individual trials. Each dot is the average of five trials. Trials during the adaptation interval (DA, 0–0.4s after transition) are shown with orange, and trials after the adaptation interval (AA, 2.0–2.4s after transition) are shown with blue. **d** Speech masking calculated based on the overlap of the spectral profile of the reconstructed speech during adaptation and the spectral profile of the reconstructed clean speech (error bars indicate standard error, *n* = 15). **e** Time course of the coefficient of determination (${{R}}^2$) of averaged reconstructed spectrograms across subjects with original noisy (yellow) and with original clean (black) spectrograms (20% cross-validations, *n* = 15). Shaded error bars indicate standard error

To illustrate the appearance of the spectral features of noise more explicitly, we averaged the reconstructed and the original spectrograms over two time intervals, during adaptation (DA, 0–0.39s after transition) and after adaptation (AA, 2–2.39s after transition), and we normalized each to its maximum value. We defined the adaptation interval for the reconstructed speech by comparing the envelope of the reconstructed and clean spectrograms (average duration = 390ms, *t*-test, *p* < 0.01). For comparison, Fig. 2a shows the average frequency power from the original spectrograms. Figure 2b (left panel) shows that the average reconstructed frequency profile during adaptation resembles the frequency profiles of the noises (*R*^2^ = 0.64 using 5 − fold cross-validation for each condition, *t*-test, *p* < 10^−6^). However, the average reconstructed frequency profile after adaptation in all three noise conditions (Fig. 2b, right panel) converges to the frequency profile of clean speech (*R*^2^ = 0.91 using 5 − fold cross-validation for each condition, *t*-test, *p* < 10^−6^). Figure 2c also shows this shift for individual trials. We quantified the time course of this effect by measuring the coefficient of determination ($R^2$) between reconstructed spectrograms with both original noisy and original clean spectrograms over time. In addition, the degree of overlap between the reconstructed spectral profile during adaptation (DA) and the spectral profile of clean speech varies across noises, as quantified by $R^2$ between reconstructed and clean speech spectrograms in Fig. 2d. The overlap was highest for the bar noise and lowest for the jet noise, meaning that during the adaptation phase, the bar noise masks the acoustic features of clean speech more than the jet noise does. This difference is a direct result of acoustic similarity between bar noise and clean speech (Supplementary Fig. 1). The $R^2$ differences over time are shown in Fig. 2e, where they show an average time of switching between the similarity of reconstructed spectrograms from noisy to clean at 420 ms (std = 70 ms). This finding shows a brief and significant decrease in the signal-to-noise ratio (SNR) of the representation of speech in the auditory cortex as the neural responses are undergoing adaptation, but the SNR is subsequently increased after the adaptation is over (analysis of individual subjects is shown in Supplementary Fig. 4).

Moreover, we confirmed that the decreased response to background noise is not due to the lack of responsiveness of electrodes to the noise stimulus relative to speech^6^, as we observed a sustained response to noise-only stimuli when it was presented to the subject without adding the foreground speech (*t*-test, *p* < 0.001, Supplementary Fig. 5). This means that the suppression of the background noise is not an inherent tuning property of the neural response to the noises and instead is contingent upon the presence of foreground speech^14,15^.

### Adaptation enhances phonetic distinctions

The reconstruction analysis showed the encoding of spectrotemporal features of the stimulus in the population neural responses. Speech, however, is a specialized signal constructed by concatenating distinctive units called phonemes, such as the /*b*/ sound in the word /*bad*/^16^. In addition, the human auditory cortex has regions specialized for speech processing that respond substantially more to speech than to other sounds^17,18^. Using a separate speech-nonspeech task, we also found many electrodes that responded significantly more to speech than to nonspeech sounds (54 out of 117 in four subjects, *t*-test, FDR corrected, *p* < 0.05, Supplementary Fig. 6). We therefore extended the spectrotemporal acoustic feature analysis to explicitly examine the encoding of distinctive features of phonemes during and after adaptation intervals.

**Fig. 3:**
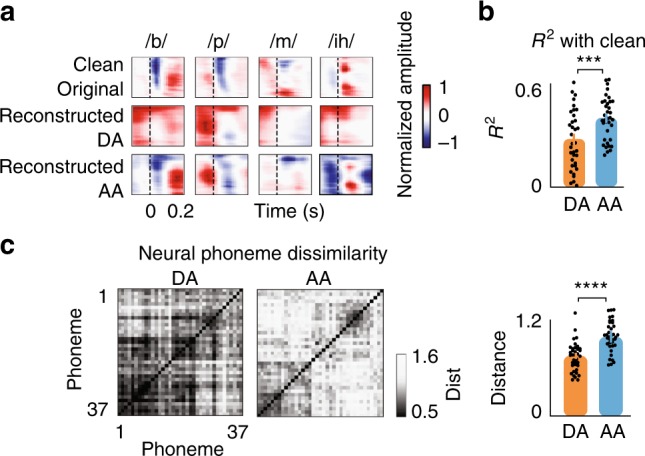
Neural degradation of phonetic features during adaptation. **a** Comparison of average phoneme spectrograms calculated from original clean spectrograms (top row), reconstructed from neural responses during adaptation (DA, middle row) and reconstructed from neural responses after adaptation (AA, bottom row). **b** Correlation between phonemes using reconstructed spectrograms and original clean spectrograms for the two intervals of during adaptation and after adaptation (mean±s.e.m. across phonemes, *N* = 37, *** paired *t*-test, *p* < 0.01). **c** Left: Phoneme dissimilarity matrices using pairwise correlation distances between phonemes for the two intervals of during adaptation and after adaptation. Right: Comparison of the average distance between phonemes during adaptation and after adaptation (mean±s.e.m. across phonemes, *N* = 37, **** paired *t*-test, *p* < 0.0001)

To examine how the cortical representation of phonetic features is affected when the background condition changes, we segmented the original and reconstructed spectrograms into individual phonemes and averaged the spectrograms of phonemes that occurred in the time intervals of during (DA) and after adaptation (AA). Figure 3a shows the original and reconstructed spectrograms of four example phonemes. The distinctive spectrotemporal features of these phonemes^16^ in reconstructed spectrograms are distorted during adaptation but are significantly enhanced afterward. For example, the phoneme /*b*/ is characterized by an onset gap followed by low-frequency spectral power. Both the gap and the low-frequency feature are masked during adaptation but are subsequently restored after adaptation. Another example is the vowel */ih/*, which is characterized by its first two formant frequencies. The frequency peaks of /*ih*/ vowels that occurred after the adaptation interval are enhanced compared to those during the adaptation interval. Quantifying the similarity of the reconstructed phoneme spectrograms during and after adaptation with the clean phoneme spectrograms shows a similar effect (Fig. 3b). Furthermore, using the high-gamma activity, we examined the relative distances between the neural representations of phonemes during and after adaptation. We generated a phoneme dissimilarity matrix^19^, which summarizes the pairwise correlation between all phoneme pairs. We found that the relative phoneme distance in the neural responses collapses during adaptation but is significantly increased after the adaptation interval (Fig. 3c). The discriminability of different reconstructed phonetic features is also reduced during adaptation to a new background condition but is increased thereafter (Supplementary Fig. 7).

**Fig. 4:**
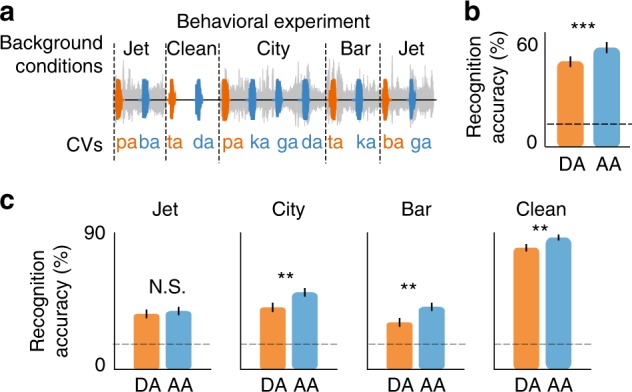
Perceptual degradation of phonetic features during adaptation. **a** Behavioral experiment designed to test the intelligibility of consonant-vowel pairs occurring during adaptation (DA, orange) and after adaptation (AA, blue) intervals. Six consonant vowels (/pa,ta,ka,ba,da,ga/) were added to the same background noise used in the main task (Fig. 1b). Half of the CVs were uttered during adaptation (DA), and the other half were uttered after adaptation (AA). **b** Comparison of correctly recognized phonemes occurring during adaptation (DA) and after adaptation (AA) intervals (mean ± s.e.m. across CVs, *N* = 1068, ** Yate’s corrected chi-square test, *p* < 0.01). **c** Comparison of correctly recognized phonemes occurring during adaptation (DA) and after adaptation (AA) intervals for each condition separately (mean±s.e.m., *N* = 267, ** Yate’s corrected chi-square test, *p* < 0.05)

Motivated by this observation, we designed a psychoacoustic task to study the perception of phonetic features during and after adaptation intervals (Fig. 4a, Supplementary Fig. 8). The task consisted of six consonant-vowel pairs (CVs, /pa,ta,ka,ba,da,ga/), chosen to cover a wide range of frequency profiles (low-frequency labials /pa,ba/, mid-frequency velar /ka,ga/, and high-frequency alveolar /ta,da/^16,20^). The CVs were embedded in the same changing background noise as the speech in the noise task. Half of the CVs were uttered during adaptation to a new background noise (DA, colored in orange), and the other half of the CVs were uttered after the adaptation interval was over (AA, colored in blue). We observed that the recognition score of the CVs during adaptation was significantly lower than that of the CVs after adaptation (Fig. 4b, Yate’s corrected chi-square test, *p* < 0.01). The match between neural (Fig. 3b) and perceptual (Fig. 4b) degradation of phonetic features during adaptation suggests an important role for neural adaptation in enhancing the discriminative features of speech that may ultimately contribute to the robust perception of speech in noise in humans^21^. Figure 4c shows the behavioral effect of adaptation in each background condition separately. The improvement in the recognition accuracy is highest for bar and lowest for the jet noise. This difference correlates well with the masking of speech features in the neural responses during adaptation to each noise (Fig. 2d). Interestingly, we also found an increase in the recognition rate after adaptation to the clean condition, meaning that phoneme recognition accuracy is also decreased immediately after the noise stops, similar to the findings of forward masking studies^22^.

### Adaptation magnitude varies across neural sites

Our analysis so far has focused on the encoding of the acoustic features of speech and noise by the population of neural sites. To examine how individual electrodes respond when the background condition changes, we first compared the magnitudes of the responses during (DA) and after adaptation (AA) by pooling electrodes across all subjects. We found variable numbers of electrodes with significant response changes during transitions to different background conditions (104 for jet, 120 for city, 122 for bar, and 78 for clean conditions, *t*-test, FDR corrected, *p* < 0.05; Supplementary Fig. 9). We also found 16 electrodes that showed no significant transient response to any of the background conditions, even though these electrodes were similarly responsive to speech (*t*-test between responses to speech vs. silence, FDR corrected, *p* < 0.01).

**Fig. 5:**
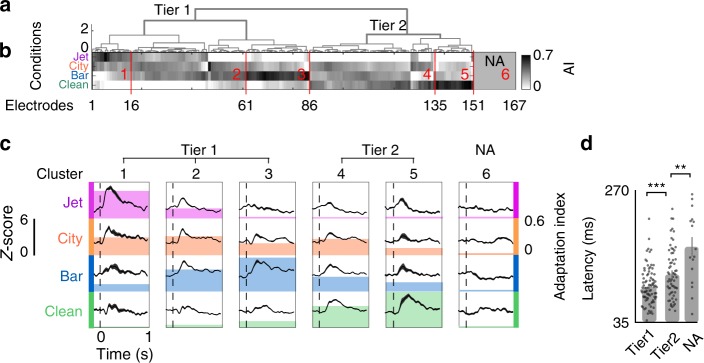
Diversity of adaptation patterns across electrodes. **a**, **b** Hierarchical clustering of adaptation indices (AI) across four background conditions. **c** Average response of electrodes in each cluster to each background condition, aligned by the time of transition. The colored backgrounds show the average adaptation indices for each cluster and condition. **d** Average latency of electrodes from the top two clusters (tier 1, tier 2) and a nonadaptive cluster (mean ± s.e.m., *N*_tier1_ = 86, *N*_tier2_ = 65, *N*_NA_ = 16, *t*-test, **p < 0.05, ***p < 0.01)

To explain the variability of adaptive response patterns across electrodes, we first defined an adaptation index (AI) as the *t*-value of a paired *t*-test between the magnitude of the responses during and after adaptation intervals. The AI for each electrode is calculated for each background condition and is normalized by subtracting the minimum over all conditions. We performed unsupervised hierarchical clustering (minimum variance algorithm, Euclidean distance) on AIs to group electrodes based on the similarity of their adaptive responses across the four background conditions (Fig. 5a). Comparison of the top two clusters of electrodes in Fig. 5b shows that the primary difference between adaptation patterns is the presence or absence of an adaptive response in transition to the clean condition (e.g., transition from speech in jet noise to clean speech). The secondary factor that further separates electrodes is their selective adaptation to different background noises. This is evident in the average responses of electrodes in each cluster, shown in Fig. 5c. For example, the first three clusters all show minimal adaptation to the clean condition but have significant adaptation to jet, city, and bar noise, respectively. Clusters 4 and 5, on the other hand, show significant adaptation to the clean condition (see Supplementary Fig. 10 for variation across subjects). Furthermore, Fig. 5d shows that the latency of the response is significantly higher for clusters of electrodes with high adaptation to clean conditions (clusters 4 and 5 in Fig. 5b) and nonadaptive clusters. Given that the response latency approximates the number of synapses between the auditory periphery and the neural site, this suggests that nonadaptive sites and sites with larger adaptation responses to clean condition are in the higher processing stages of the auditory pathway.

### Adaptation is unaffected by the attentional focus

**Fig. 6:**
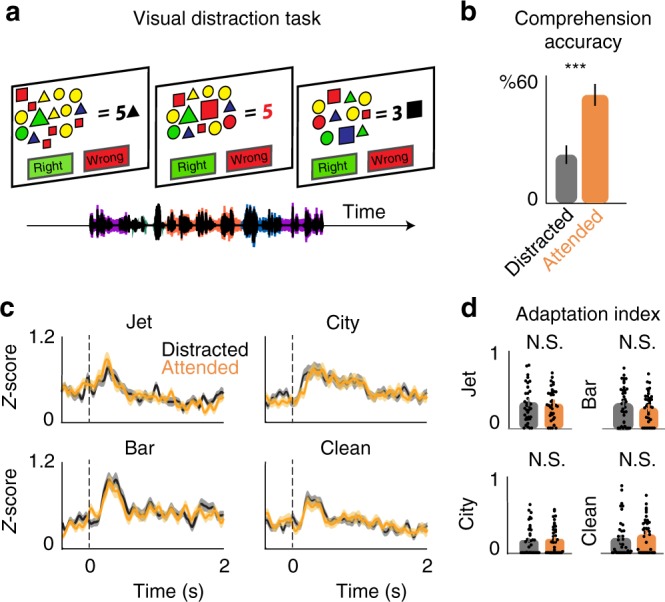
The effect of attention on neural adaptation. **a** Schematic of the visual distraction task performed to divert the attention of subjects from the noisy speech task. **b** Subjects’ behavioral response accuracy in response to the contextual questions from speech stories in two conditions of Distracted (Black) and Attended (orange) (mean ± s.e.m., *N*_distracted_ = 48, *N*_attended_ = 96, ***Yate’s corrected chi-square test, *p* < 0.01). **c**, **d** Comparison of adaptation patterns and adaptation indices in response to the noisy speech task for the two conditions of Distracted and Attended (mean ± s.e.m. across electrodes, *N* = 37)

To examine the effect of subject’s attention on adaptive responses to background noise changes, we engaged the subjects in a demanding visual task as they heard the speech in noise sounds (Fig. 6a). We then repeated the speech in noise task without the visual task and asked the subjects to attend to speech instead. Figure 6b shows a significant difference between the speech comprehension accuracy with and without the secondary visual task and confirms the efficacy of the secondary task in distracting the subjects from the auditory task (Fig. 6b). Despite the large difference between the attentiveness of the subjects to the auditory task in these two experimental conditions (Yate’s corrected chi-square test, p < 0.001), we did not observe a significant difference between the neural responses to speech and the adaptation patterns with and without the secondary visual task (Fig. 6c and d, individual subjects in Supplementary Fig. 11). The similarity of responses in the two attention conditions suggests that adaptation to changing background noise may primarily be a bottom-up phenomena^3,4^.

### Spatial organization of the adaptation patterns

**Fig. 7:**
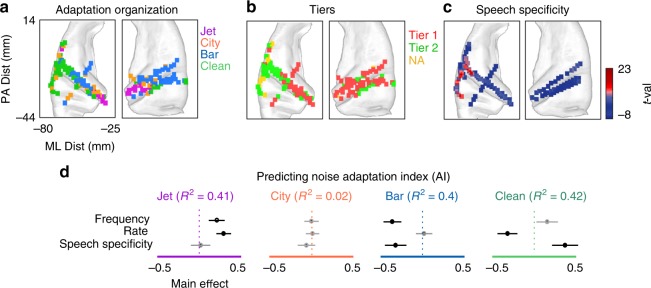
Spatial organization of adaptation patterns. **a** Anatomical organization of electrodes with the largest adaptation to each background condition on the left and right brain hemispheres. **b** Anatomical organization of the top two clusters and nonadaptive electrodes. **c** Anatomical organization of speech specificity based on *t*-values of an unpaired *t*-test between neural responses to speech and nonspeech sound categories. **d** R-squared values for predicting the adaptation indices from electrodes’ best frequency, best rate (temporal modulation), and speech specificity. The contribution (main effect) of each parameter in predicting the adaptation indices is also shown (*t*-test, black: *p* < 0.01, gray: *p* > 0.05)

We examined the spatial organization of the adaptive responses to different background conditions. Figure 7a shows the spatial organization of AIs for jet, city, bar, and clean conditions on the average brain MRI (FreeSurfer template brain, ICBM152). Each pixel in Fig. 7a is a 2mm × 2mm square, and the color of the square at each location is chosen based on the maximum AI at that location across the four background conditions (AIs of individual electrodes are shown in Supplementary Fig. 12). Figure 7a shows that adaptation to jet noise is strongest in the medial (deep) electrodes on both hemispheres, while adaptation to bar noise is stronger in the lateral (superficial) electrodes. The spatial organization of adaptive responses shown in Fig. 7a is largely due to the spatial organization of tuning properties (Supplementary Fig. 13). Furthermore, an intriguing observation from Fig. 7a is that electrodes with the largest adaptation to the clean condition are mostly located in the STG and in the left brain hemisphere. The spatial organization of the two tiers from the unsupervised clustering in Fig. 5a is also consistent with the spatial organization of the adaptation to the noises and to the clean because tier two mostly consists of electrodes that show the strongest adaptation in transition to the clean condition (Fig. 7b). Moreover, the stronger adaptation to the clean condition in higher-level cortical areas, such as the STG, is highly correlated with the spatial organization of speech specificity of electrodes (Fig. 7c, *r* = 0.51, $p < 10^{ - 9}$).

To study why neural sites adapt differently to the background conditions, we examined the relationship between adaptation patterns and both the spectrotemporal tuning and the speech specificity of electrodes. We characterized the tuning properties of an electrode by calculating its spectrotemporal receptive field (STRF)^23^. We measured two parameters from each STRF to describe the electrodes’ preferred frequency (best frequency) and preferred temporal modulation (best rate). The best-frequency parameter differentiates tuning to high versus low acoustic frequencies and is defined as the spectral center of the excitatory region of the STRF. The best-rate parameter is measured from the modulation transfer function^24^ (Supplementary Fig. 14) and differentiates tuning to slow and fast acoustic features. In addition, we also measured the degree of speech specificity of the electrodes, defined as the *t*-value of a paired *t*-test between the responses of each electrode to speech and nonspeech sounds (see supplementary Fig. 6 for the list of nonspeech sounds).

To study the contribution of each tuning dimension in predicting how an electrode responds in transition to a particular background condition, we used linear regression to predict AIs from the tuning parameters. Figure 6d shows the predictive power for each tuning parameter and the overall correlation between the actual and predicted AIs of each background condition. The AIs of all conditions except city (the least stationary noise, Supplementary Fig. 1) are highly predictable from electrodes’ response properties ($R_{jet}^2 = 0.41,\,p < 10^{ - 12};\,R_{city}^2 = 0.02, p < 0.01;\,R_{bar}^2 = 0.4,p < 10^{ - 12};\, R_{clean}^2 = 0.42,\,p < 10^{ - 12}$). Figure 7d also shows that electrodes with tuning to higher frequencies also show higher adaptation to the high-frequency jet noise (positive main effect, 0.27). On the other hand, lower-frequency neural sites show higher adaptation to low-frequency bar noise (negative main effect, −0.46, *t*-test, *p* < 0.001). Temporal modulation tuning of electrodes is positively correlated with the AI of jet noise (positive main effect, 0.38, *t*-test, *p* < 0.001), which is also the condition with fastest temporal modulation (Supplementary Fig. 1). Temporal modulation (rate) is negatively correlated with the AI of the clean condition (negative main effect, −0.47, *t*-test, *p* < 0.001), meaning that electrodes with a longer temporal integration window had the highest adaptive response in transition to the clean condition. The speech specificity of electrodes was positively correlated with the AI of the clean condition (positive main effect, 0.48, *t*-test, *p* < 0.001), indicating that the electrodes that show the highest adaptation in transition from noisy to clean speech are the ones that also respond more selectively to speech over nonspeech sounds. Together, these results show that the adaptation patterns across electrodes are largely predictable from the response properties of those electrodes, such that electrodes that are tuned to the acoustic properties of a background condition also show the strongest adaptation to that condition.

## Discussion

We examined the dynamic reduction in background noise in the human auditory cortex using invasive electrophysiology combined with behavioral experiments. We found that when a new background noise appears in the acoustic scene during speech perception, the auditory neural responses momentarily respond to noise features, but rapidly adapt to suppress the neural encoding of noise, resulting in enhanced neural encoding and perception of phonetic features of speech. We found a diversity of adaptation pattern across electrodes and cortical areas, which was largely predictable from the response properties of electrodes. Moreover, adaptation was present even when the attention of the subjects was focused on a secondary visual task.

Previous studies have shown that the auditory cortex in animals and humans encodes a noise-invariant representation of vocalization sounds^3–9,25^. Our study takes this further by examining the dynamic mechanisms of this effect and how they change the representation of the acoustic scene as adaptation unfolds. Our finding of reduced neuronal responses to noise is consistent with studies that propose adaptation as an effective coding strategy that results in an enhanced representation of informative features when the statistical properties of the stimulus change^26–28^. Although the adaptive encoding of a particular stimulus dimension has been shown in several subcortical^29–32^ and cortical areas^3,4^, our study goes further by identifying the specific acoustic features of speech and background noise that are encoded by the neural responses over the time course of adaptation.

We found that the magnitude of adaptation to different background noises varied across neural sites, yet it was predictable from the spectrotemporal tuning properties of the sites. This observation was made possible by the sharp spectral contrast between the three background noises used in our study. This means that the neural sites whose spectral tuning match the spectral profile of a particular noise also have a stronger adaptive response to that noise. We also found a population of neural sites that did not show any adaptation to the noises in our study, which could be due to the sparse sampling of the spectrotemporal space caused by the limited number of noises we used. In addition to the spectral overlap, previous studies have shown that separating an auditory object from a background noise that has a temporal structure requires integration over time^33,34^. Experiments that systematically vary the temporal statistics of the background noise^35^ are needed to fully characterize the dependence of adaptation on the statistical regularity and the history of the stimulus^36^.

We found that adaptation in transition from noisy to clean speech occurred only in higher cortical areas, such as in the left-hemisphere STG. While previous studies have already established the specialization of the STG for speech processing^17,18^, our finding uncovers a dynamic property of this area in response to speech. The magnitude of the adaptive response in transition to the clean condition was highly predictable from the speech specificity of electrodes, which is a nonlinear tuning attribute. It is worth mentioning that these sites were also highly responsive to foreign languages that were incomprehensible to the subjects. Therefore, the speech specificity of neural sites in our study is likely caused by tuning to speech specific spectrotemporal features and not by higher order linguistic structures^37^. The transient response to the clean condition observed in the speech-specific electrodes may indicate adaptation of these sites to the unmasked features of speech, which reappear when the noise stops and indicate the recovery of speech-selective responses from their noise-adapted state^38^. This result is also consistent with studies of the neural mechanism of forward masking, which has been reported in the auditory periphery^39^ and the auditory cortex^38^, where the neural response to a clean target sound changes depending on the sound that preceded the target.

Using a behavioral paradigm, we show that the recognition of phonemes is degraded during the adaptation interval to a new background condition. Moreover, we found that the decrease in the phonetic feature recognition was greater when transitioning to a background noise that overlaps spectrally with speech, such as in the case of bar noise. This reduced phoneme recognition accuracy was consistent with the observed degradation of the phoneme representation in the neural data. This finding confirms the role that adaptation plays in enhancing the signal contrast with the background^40^, which results in an improved identification of its distinct features that are relevant for perception. Interestingly, we also observed a reduced behavioral accuracy in the perception of the phonemes when transitioning from a noisy background to the clean condition. This behavioral observation is consistent with the psychophysical studies of forward masking, where the detection of a target sound can be impaired by the preceding sound^22^, particularly when the acoustic properties of the noise and target overlap^41^.

We found that the strength of adaptation to background noises was stronger when listening to speech in noise compared to listening to noise alone. This means that the presence of speech was necessary for the observed suppression of noise features in the neural responses. The representation of speech in the human auditory cortex is also modulated by top-down signals, including the semantic context^42–45^ and attention^46–48^. It was therefore plausible that a momentary lapse in the subjects’ attention at the point of background switch could cause the transient neural responses we observed. Controlling for this possibility, we found that adaptation results are equally present even when the attention of the subject was directed towards a demanding secondary visual task. Although the behavioral performance of the subject during the auditory task significantly decreased with the added visual task, there was no detectable difference in adaptation patterns in the two experimental conditions. Moreover, While we used speech stories in the native language of the subjects, our behavioral experiment showed a decrease in phoneme recognition accuracy even when nonsense speech (CVs) was used, suggesting that the enhanced effect of adaptation exists independent of linguistic context^37,45^. As a result, the adaptation results we observed are likely due to bottom-up nonlinear mechanisms such as synaptic depression^4,49^ and divisive gain normalization^3,50^. These mechanisms can separate an acoustic stimulus with rich spectrotemporal content, such as speech, from the more stationary noises that are commonly encountered in naturalistic acoustic environments^4,6,7^.

In summary, our findings provide insights on the dynamic and adaptive properties of speech processing in the human auditory cortex that enables a listener to suppress the deleterious effects of environmental noise and focus on the foreground sound, therefore making speech a reliable and robust means of communication.

## Methods

### Intracranial recordings

Eight adults (five females) with pharmacoresistant focal epilepsy were included in this study. Subjects 1 to 6 were presented with the complete noisy speech task (Figs 1–5, 7). Subjects 7 and 8 were presented with the visual distraction task (Fig. 6). All subjects underwent chronic intracranial encephalography (iEEG) monitoring at North Shore University Hospital to identify epileptogenic foci in the brain for later removal. Six subjects were implanted with stereo-electroencephalographic (sEEG) depth arrays, one with grids and strip arrays, and one subject with both (PMT, Chanhassen, MN, USA). Electrodes showing any sign of abnormal epileptiform discharges, as identified in epileptologists’ clinical reports, were excluded from the analysis. All included iEEG time series were manually inspected for signal quality and were free of interictal spikes. All research protocols were approved and monitored by the institutional review board at the Feinstein Institute for Medical Research, and informed written consent to participate in research studies was obtained from each subject before implantation of electrodes.

Intracranial EEG (iEEG) signals were acquired continuously at 3kHz per channel (16-bit precision, range±8mV, DC) with a data acquisition module (Tucker–Davis Technologies, Alachua, FL, USA). Either subdural or skull electrodes were used as references, as dictated by recording quality at the bedside after online visualization of the spectrogram of the signal. Speech signals were recorded simultaneously with the iEEG for subsequent offline analysis. The amplitude of the high-gamma response (75–150 Hz) was extracted using the Hilbert transform^51^ and was resampled to 100 Hz. The high-gamma responses were normalized based on the responses recorded during a 2-min silent interval before each recording.

### Brain maps

Electrode positions were mapped to brain anatomy using registration of the postimplant computed tomography (CT) to the preimplant MRI via the postop MRI^52^. After coregistration, electrodes were identified on the postimplantation CT scan using BioImage Suite^53^. Following coregistration, subdural grid and depth electrodes were snapped to the closest point on the reconstructed brain surface of the preimplantation MRI. We used the FreeSurfer automated cortical parcellation^54^ to identify the anatomical regions in which each electrode contact was located within ~3mm resolution (the maximum parcellation error of a given electrode to a parcellated area was <5voxels/mm). We used Destrieux’s parcellation, which provides higher specificity in the ventral and lateral aspects of the medial lobe^55^. Automated parcellation results for each electrode were closely inspected by a neurosurgeon using the patient’s coregistered postimplant MRI.

### Stimulus and auditory spectrogram

Speech material was short stories recorded by four voice actors (two male and two female voice actors; duration: 20 min, 11025 Hz sampling rate). The three noises were taken from the NOISEX-92 corpus^56^. Different three- or six-second segments of the noise were chosen randomly for each transition and were added to the speech at a 6 dB signal-to-noise ratio (noisy speech task). The SNR of 6 dB was chosen to ensure the intelligibility of foreground speech^57^. In three of the subjects, we ran an additional task after the adaptation task, where they listened to the same speech utterances without the additive noises (clean speech task).

All stimuli were presented using a single Bose SoundLink Mini 2 speaker situated directly in front of the subject. To reduce the inevitable acoustic noise encountered in uncontrolled hospital environments, all electrical devices in patients’ room were unplugged except the recording devices and the door and windows were closed during the experiment to prevent interruption. We also recorded the clean speech task without the noise in three of the subjects for direct comparison of neural responses in the same hospital environment. Speech volume was adjusted to a comfortable listening volume.

The time-frequency representation of speech sounds was estimated using a model of cochlear frequency analysis, consisting of a bank of constant 128 asymmetric filters equally spaced on a logarithmic axis. The filter bank output was subjected to nonlinear compression, followed by a first order derivative along spectral axis (modeling inhibitory network), and finally an envelope estimation operation. This resulted in a two dimensional representation simulating the pattern of activity on the auditory nerve^24^. The Matlab code to calculate the auditory spectrogram is available at https://isr.umd.edu/Labs/NSL/Software.htm. The output of the filter bank was then resampled to 13 bands.

### Speech-specificity task

To quantify the speech specificity of each neural site, four of the subjects (subjects 1, 2, 4, and 6) performed the speech-nonspeech task. Subjects listened to 30 min of audio containing 69 commonly heard sounds (Supplementary Fig. 6). The sounds consisted of coughing, crying, screaming, different types of music, animal vocalization, laughing, syllables, sneezing, breathing, singing, shooting, drum playing, subway noises, and speech by different speakers. To determine the speech-specificity index, we first normalized the response of each site using the mean and variance of the neural data during the silent interval. We then averaged the normalized responses over the presentation of each sound. Finally, we performed an unpaired *t*-test between the averaged responses of all speech and all nonspeech sounds to obtain a t-value for each site denoting the selectivity to speech over nonspeech sounds.

### Visual attention task

To control for the effect of attention on the adaptation patterns, we designed a visual experiment which we tested on two subjects (subject 7 and 8). We used 10 min of the adaptation task and presented it in two conditions to the subject: I) when the subject was engaged in the visual task (auditory distracted) and II) without the visual task (auditory attended). The subjects were presented with the distracted condition first where they were asked to perform a visual search task and ignore the sound (the speech in noise task) that was presented simultaneously. The visual search task was a two-choice test. The subjects had a maximum of seven seconds to answer each question and had to count either the number of colors (Question 1) or the number of shapes (Question 2) and respond whether the answer shown on the screen was right or wrong. In the visual condition, subject 1 could answer 132 questions in 10 min with 74% accuracy, and subject 2 could answer 164 questions in 10 min with 87% accuracy. In the Attended experimental condition, the subject attended to the sound (the adaptation task) without the visual secondary task. To control for the possible confounding effect of visual stimulus in the distracted experimental condition, we asked the subject to fixate on the visual search task while different questions were shown, but the subject was not required to answer any of those questions. To measure the efficacy of the visual task in engaging the attention of the subject, at the end of each experimental block we asked the subjects contextual questions about the speech stories. The subject had three options: (1) Right, (2) Wrong and (3) Unsure. The total number of questions was 72.

### Behavioral task

12 subjects (seven males, five females) with self-reported normal hearing participated in this experiment. The task consisted of six consonant-vowel pairs (CVs, /pa,ta,ka,ba,da,ga/) spoken by two male and two female speakers (a total of 24 tokens). The tokens were embedded in changing background noise identical to the main speech in the noise experiment shown in Fig. 1b. Half of the CVs were uttered immediately after the transition to a new background noise (during adaptation, DA), and the other half of the CVs were uttered 1.5 s after transition (after adaptation, AA). Noises were added to CVs at SNR of −4 dB. The task was presented to the subjects using Matlab. The participants responded via a multiple-choice graphical user interface (GUI) in Matlab that included the six CVs in addition to an unsure option. Subjects were required to report the CV continually and were all able to keep up with the rapid speed of CV presentation. All subjects provided written informed consent. The Institutional Review Board (IRB) of Columbia University approved all procedures.

### Stimulus reconstruction

We used a linear model to map the neural responses (R) to the auditory stimulus (S). We trained the model on clean speech that was played to the subject after the noisy speech experiment. We used time lags from −250 to 0 ms of the neural data as the input to the ridge regression (R). The model (g) is calculated by minimizing the MSE between reconstructed and original spectrograms, which results in the cross-correlation of the stimulus and the ECoG data normalized by the autocorrelation of the ECoG data.

We then applied the model to the noisy neural data. For the analyses shown in Figs 1 and 2, we first generated the reconstruction model for each subject individually and then averaged the reconstructed spectrograms across subjects^58^.

### Spectrotemporal receptive fields

STRFs were computed by normalized reverse correlation algorithm^59^ using STRFLab^59^. Regularization and sparseness parameters were found via cross-validation. The best-frequency and response latency parameters were estimated by finding the center of the excitatory region of STRF along frequency and time dimensions. The best-rate parameter was estimated from the 2-dimensional wavelet decomposition of the STRF^24,60^. The wavelet decomposition extracts the power of the filtered STRFs at different temporal modulations (rates)^24,60^. The modulation model of STRFs has four dimensions: scale, rate, time, and frequency. To estimate rate, we first averaged the model over three dimensions of time, frequency, and scale to calculate a rate vector. Next, we found the weighted average of the rate vector, where weights are the rate values.

### Phoneme responses

We segmented the speech material into time-aligned sequences of phonemes using the Penn Phonetics Lab Forced Aligner Toolkit^61^, and the phoneme alignments were then manually corrected using Praat software^62^. The spectrograms were aligned to the onset of phonemes with a time window of 200 ms. To minimize the preprocessing effects, we did not normalize the natural variation in phoneme length. The phoneme pairwise distances were calculated based on the Euclidean distance between each pair of phonemes.

### Calculating adaptation indices

To characterize the adaptation index (AI), we measured the t-value of a paired *t*-test between the neural response of each neural site in time intervals of 0 to 0.7 s (during adaptation, DA) and 2 to 2.7 s (after adaptation, AA) after the transition to each background condition (time 0). AIs were normalized by subtracting the minimum over all conditions, followed by a division by their sum.

### Reporting Summary

Further information on research design is available in the Nature Research Reporting Summary linked to this article.

## Supplementary information

Supplementary Information

Reporting Summary

## Data Availability

The data that support the findings of this study are available on request from the corresponding author [N.M.]. A reporting summary for this Article is available as a Supplementary Information file.

## Code Availability

The codes for performing phoneme analysis, calculating high-gamma envelope, and reconstructing the spectro gram are available at *http://naplab*.*ee*.*columbia*.*edu/naplib*.*html*^63^.
