# Supplementary material for: Adaptation of the human auditory cortex to changing background noise
Source: Nat Commun. 2019 Jun 7;10:2509. doi: 10.1038/s41467-019-10611-4 (PMC6555798; doi:10.1038/s41467-019-10611-4)
Supplement: Supplementary file 1 — Supplementary Information [file 41467_2019_10611_MOESM1_ESM.pdf]

Supplementary Information for

Adaptation of the human auditory cortex to changing background noise

Bahar Khalighinejad[1,2], Jose L. Herrero[3,4], Ashesh D. Mehta[3,4], Nima Mesgarani[1,2]

[1]*Mortimer B. Zuckerman Mind Brain Behavior Institute, Columbia University, New York, NY*

[2]*Department of Electrical Engineering, Columbia University, New York, NY*

[3]*Hofstra Northwell School of Medicine, Manhasset, NY, United States*

[4]*The Feinstein Institute for Medical Research, Manhasset, NY, United States*

correspondence to: nima@ee.columbia.edu

This PDF file includes:

Figs. S1 to S14

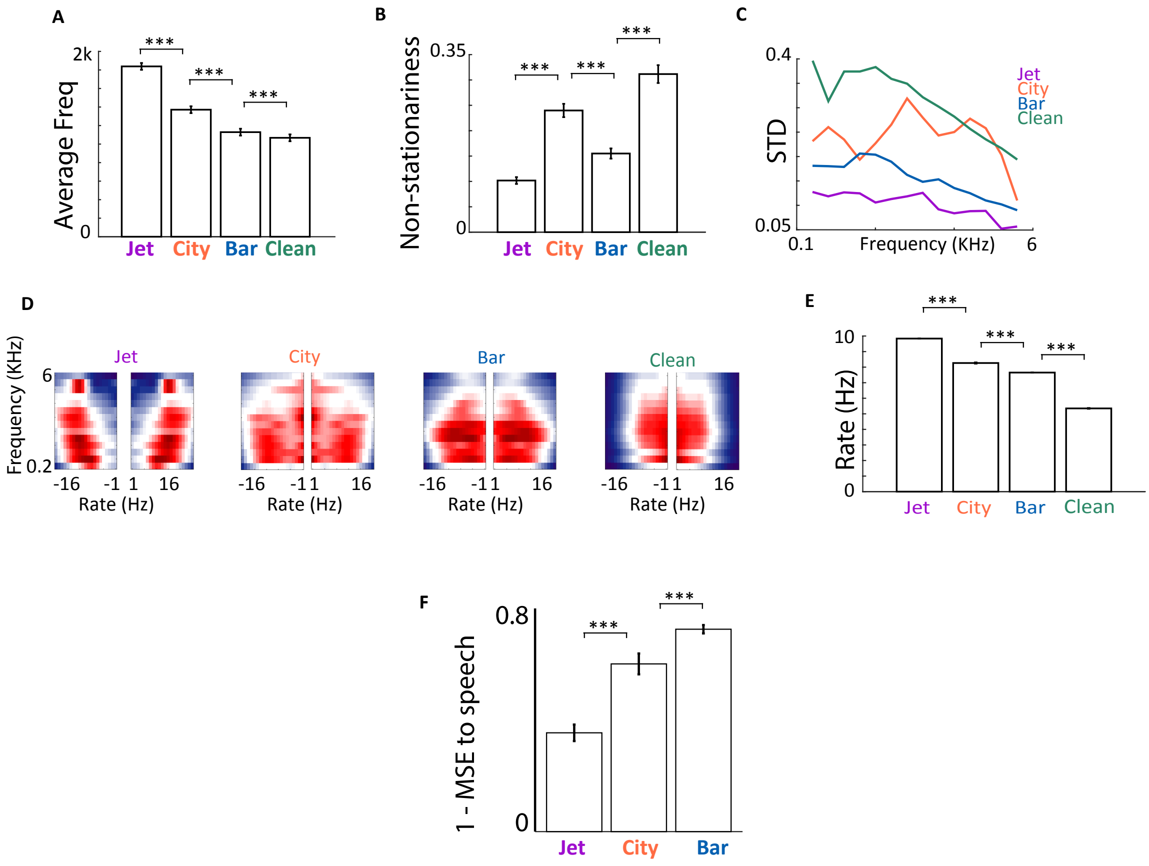

Supplementary Fig. 1. Spectrotemporal properties of each background condition

A) The weighted average (center of gravity) of frequencies for four conditions of jet, city, bar and clean computed from the acoustic spectrograms.

Average Frequency of Jet > City > Bar> Clean

B) We defined the non-stationariness by finding the standard deviation of each frequency band over time and then averaging across frequencies (*1*). The higher standard deviation indicates higher nonstationarity of the condition. The difference between stationarity of the conditions is significant and varies in the following order:

stationariness *of Jet > Bar > City > Clean speech*

C) Nonstationarity across different frequency bands.

D) Frequency-rate model of each of the background conditions and clean speech.

E) Jet has the highest rate across conditions, and clean has the lowest rate.

F) We computed the degree of speech masking for each condition by calculating the overlap between the frequency profile of each noise and clean speech:

$$masking_{bar} > masking_{city} > masking_{jet}$$

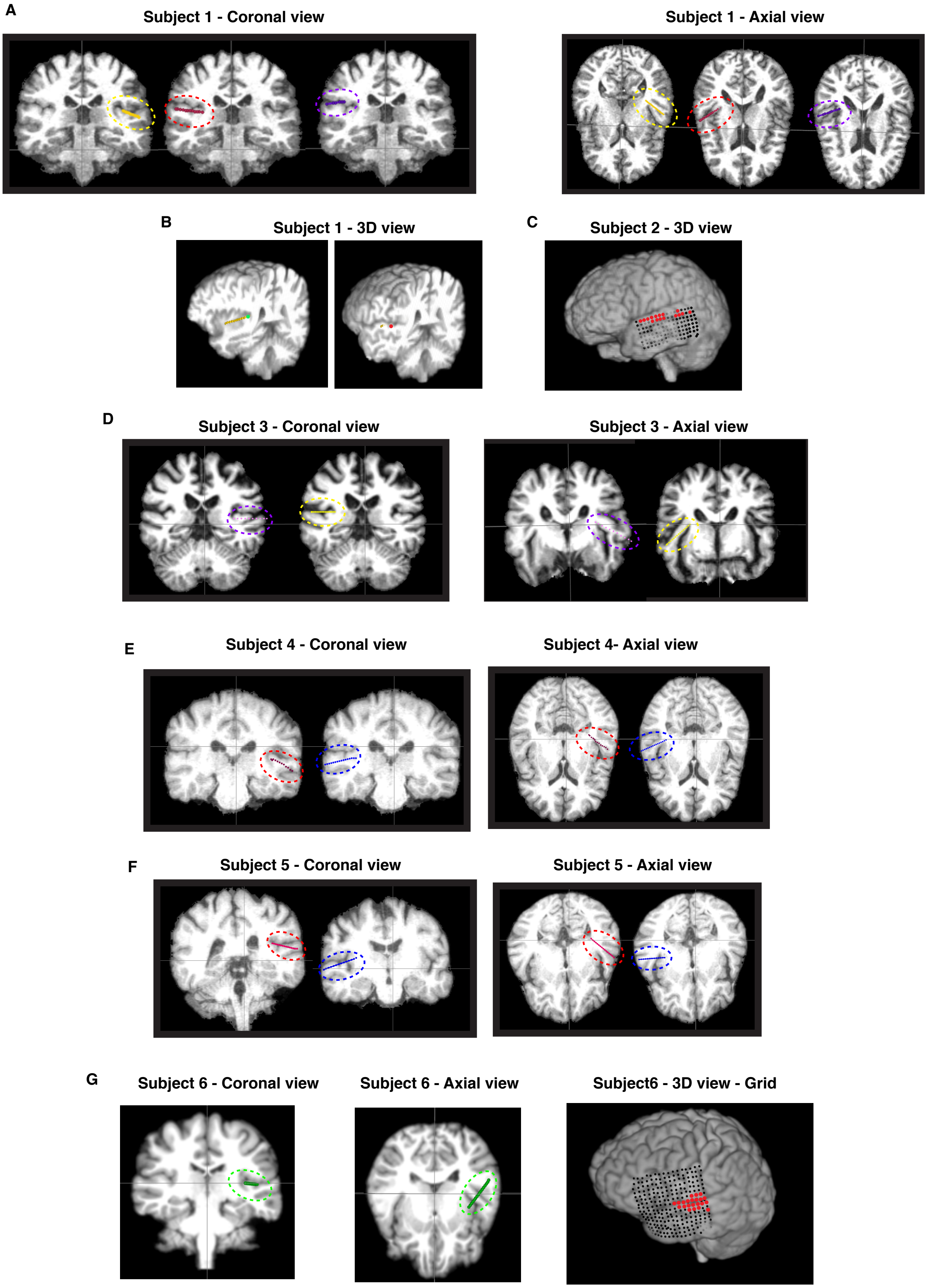

Supplementary Fig. 2. Location of grid and depth electrodes on MRI of each individual subject. Subjects 1, 3, 4 and 5 have bilateral depth electrodes. Subject 2 has only grid on the left hemisphere. Subject 6 has both grid and depth electrodes only on the left hemisphere.

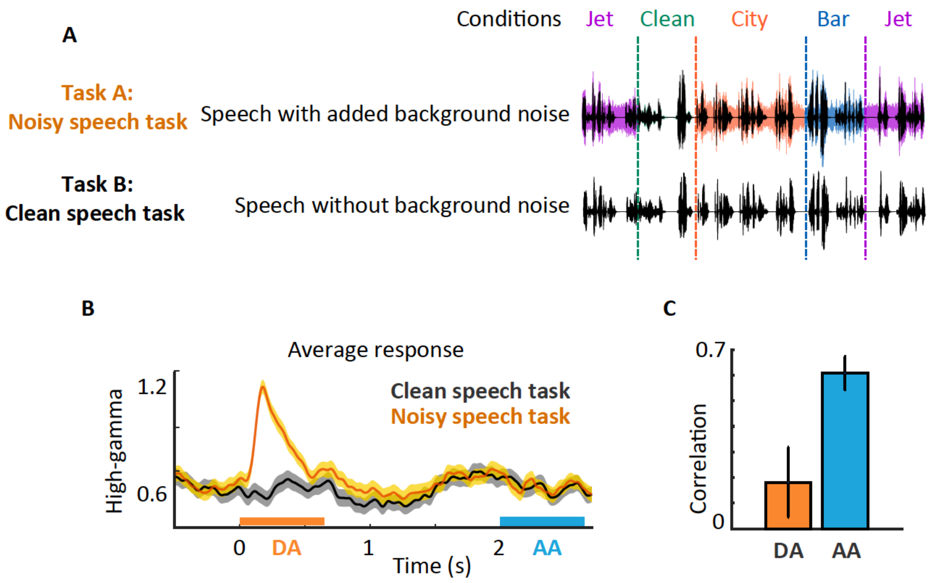

Supplementary Fig. 3. Schematics of the speech in noisy and clean speech tasks and the adaptation interval for neural responses.

A) Schematic of the speech in noise and clean speech tasks that were presented to the subjects.

B) The average neural response time-aligned by transition to a new background condition in noisy speech task (orange) and the average neural response constructed similarly but from the clean speech task (black). The average adaptation time for neural responses is 670 ms.

C) Comparison of Pearson correlation coefficient between the responses to clean speech task and noisy speech task for two time intervals of during adaptation (DA) and after adaptation (AA).

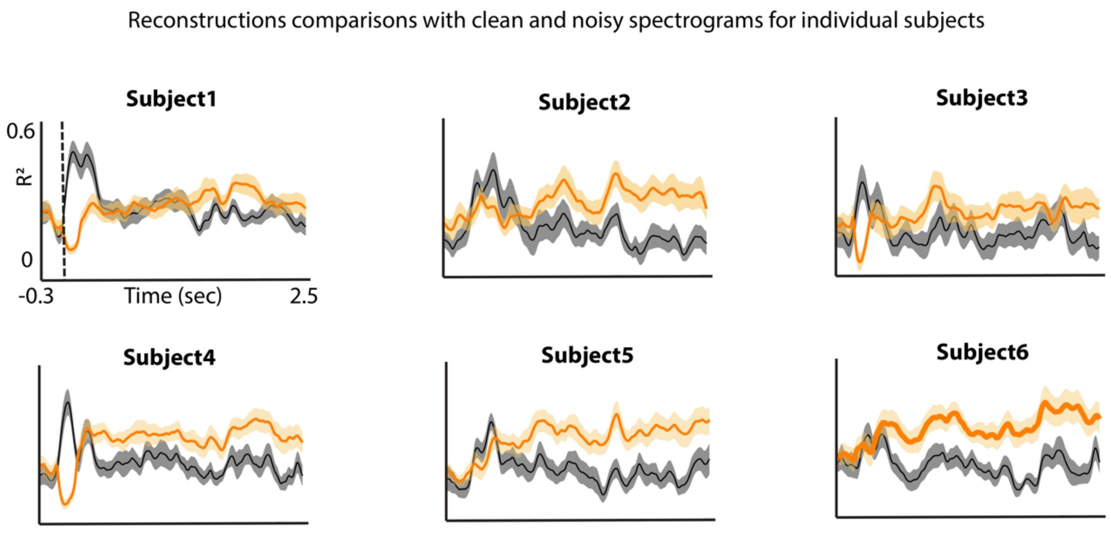

Supplementary Fig. 4. Comparison of reconstructed spectrograms with respect to clean and noisy spectrograms for individual subjects

Time course of the coefficient of determination (R^2) of reconstructed spectrograms with respect to (wrt) original noisy (orange) and with respect to original clean (black) spectrograms (20% cross-validations, n = 15) for individual subjects.

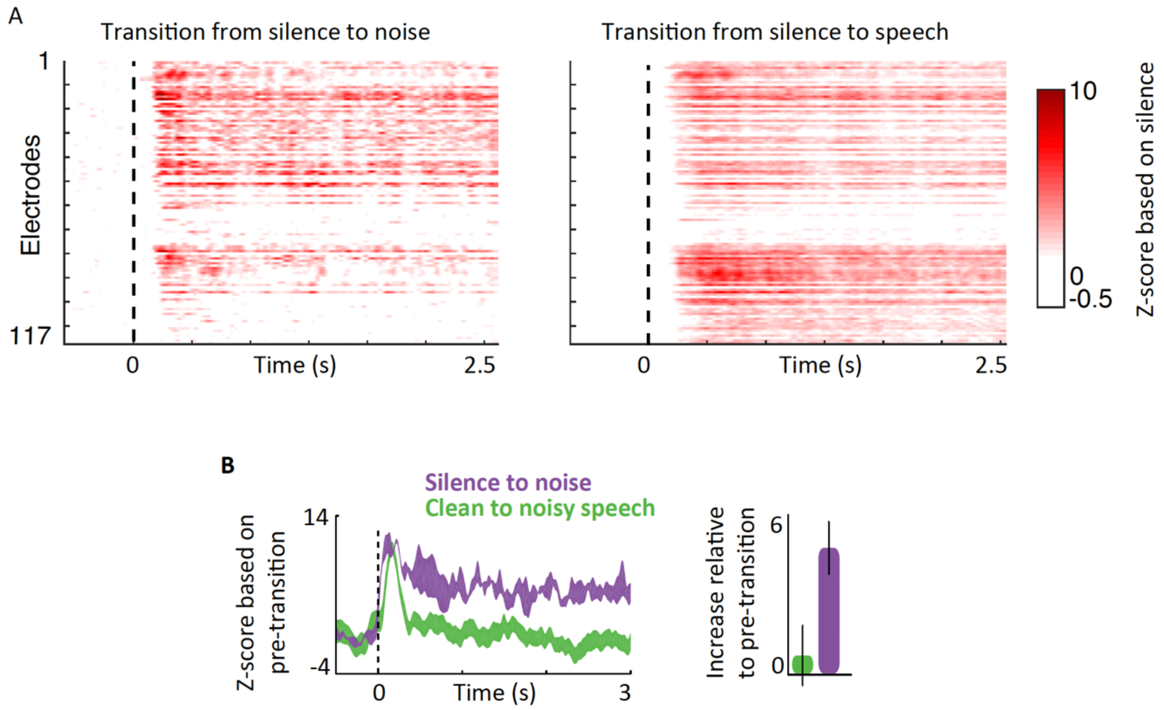

Supplementary Fig. 5. The neural response to the noise and speech alone.

A) We played the noises and speech separately in four of the subjects (117 electrodes). The goal of this experiment was to test whether the adaptation patterns are caused by the non-responsiveness of electrodes to noise stimulus relative to speech. We observed that unlike the noisy speech task, the neural responses to the noise stimuli continued over time.

B) Left: Comparison of the average neural response when transitioning from silence to noise-only stimulus (purple) with the average neural response when transitioning from clean speech to speech in noise (green). Neural responses are normalized based on pre-transition intervals. Right: comparison of the baseline change after adaptation interval (2 to 3 sec after transition) relative to the pre-transition interval (-0.5 sec to 0 sec) in the two conditions.

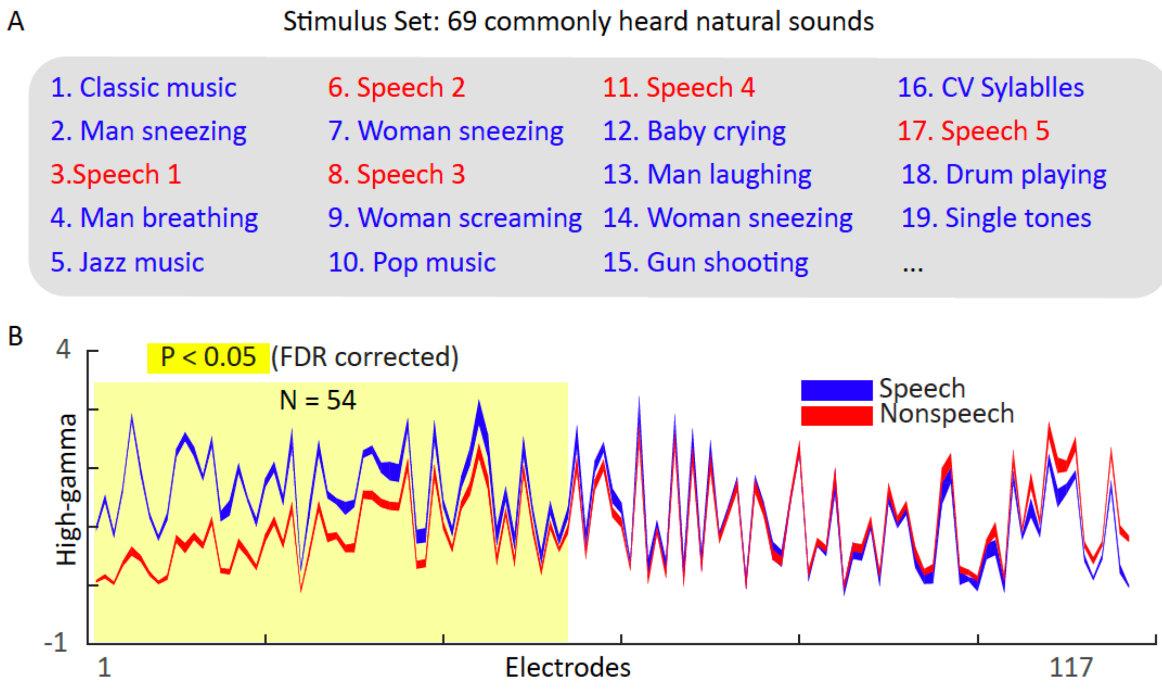

Supplementary Fig. 6. Speech-specificity task and the speech-specific electrodes.

Out of 117 electrodes, 54 (45%) responded significantly more to speech over nonspeech sounds (*speech-specific* electrodes). The remaining 55% of electrodes responded equally to both speech and nonspeech sounds (*speech-nonspecific* electrodes). The majority of speech-specific electrodes were located on the left superior temporal gyrus, the same area that showed a stronger transient response when the background noise stopped.

The complete list of categories of nonspeech sounds is as follows:

1. Coughing
2. Crying
3. Screaming
4. Music (Jazz, Pop, Classical)
5. Animal vocalization
6. Laughing
7. Syllables
8. Sneezing
9. Breathing
10. Singing
11. Shooting
12. Tones
13. Drum playing
14. Subway noise

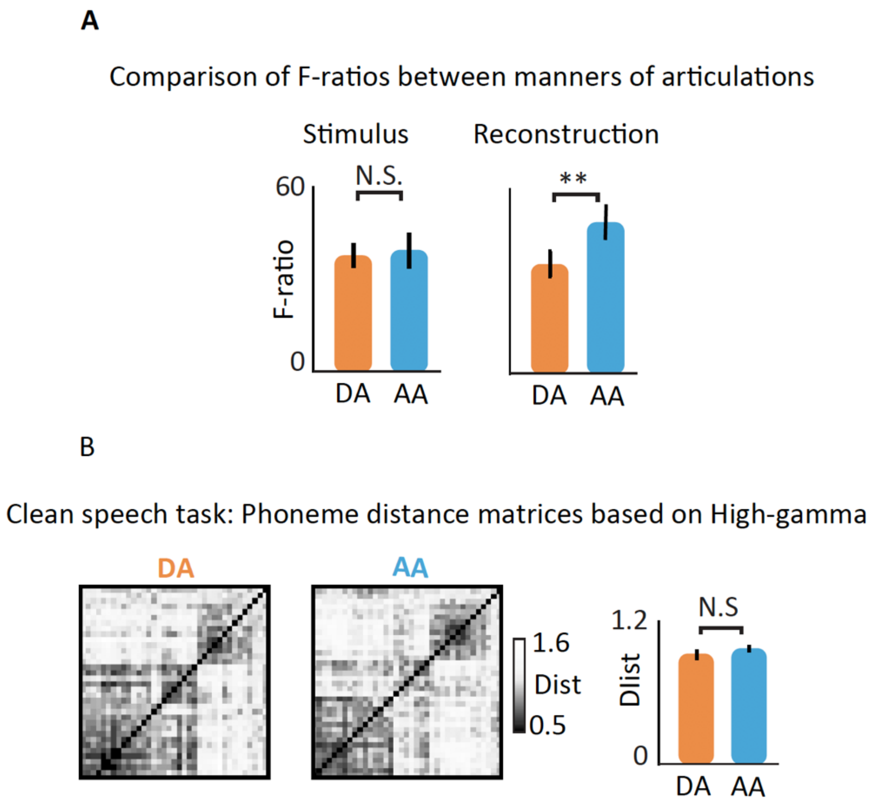

Supplementary Fig. 7. The discrimination between phonemes.

A) We quantified the discriminability of all phonemes using the ratio of between-group to within-group variability (F-statistics) for five phonetic features of manners of articulation (plosives, fricatives, vowels, nasals, and approximants) (*2–4*).

B) We performed the same analysis as Fig. 3B in neural responses to speech without the changing background conditions, and we did not observe a suppression in pairwise distances of phonemes.

A

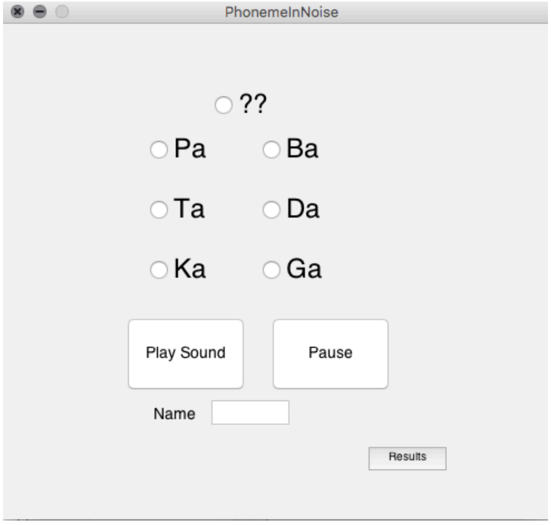

B

Subject	During adaptation	After adaptation
1	0.6000	0.6900
2	0.5700	0.6500
3	0.5000	0.5600
4	0.5600	0.6100
5	0.6500	0.7100
6	0.4800	0.6200
7	0.3100	0.3900
8	0.5600	0.7100
9	0.1458	0.2188
10	0.1649	0.1856
11	0.5200	0.6400
12	0.5500	0.6200

Supplementary Fig. 8. The psychoacoustic task to study the perception of phonetic features.

A) The task consisted of 200 phonemes randomly assigned to the during adaptation (DA) and after adaptation (AA) intervals. Subjects had to click on the consonant-vowel pair (CV) that they perceived as soon as the CV ends. The time interval between each CV was 1.5 s.

B) The recognition rate during adapation (DA) and after adaptation (AA) for each subject individually.

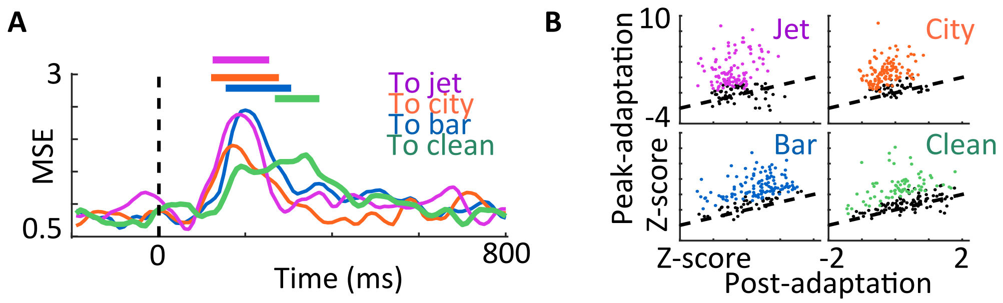

Supplementary Fig. 9. Comparison of adaptation time and electrodes for different background conditions

A) Mean squared error (MSE) between the high-gamma responses to clean and noisy speech is shown over time. The horizontal bars on top of the plot highlight the time points where at least 25% of electrodes showed a significant difference between the two conditions (corrected for false discovery rate, q<0.01).

B) The distortion effect on single electrodes for each of the conditions. The x-axis shows the z-scored high-gamma response after adaptation, the y-axis shows the z-scored high-gamma response during adaptation intervals, and electrodes with significant distortion are colored (paired t-test, p<0.01).

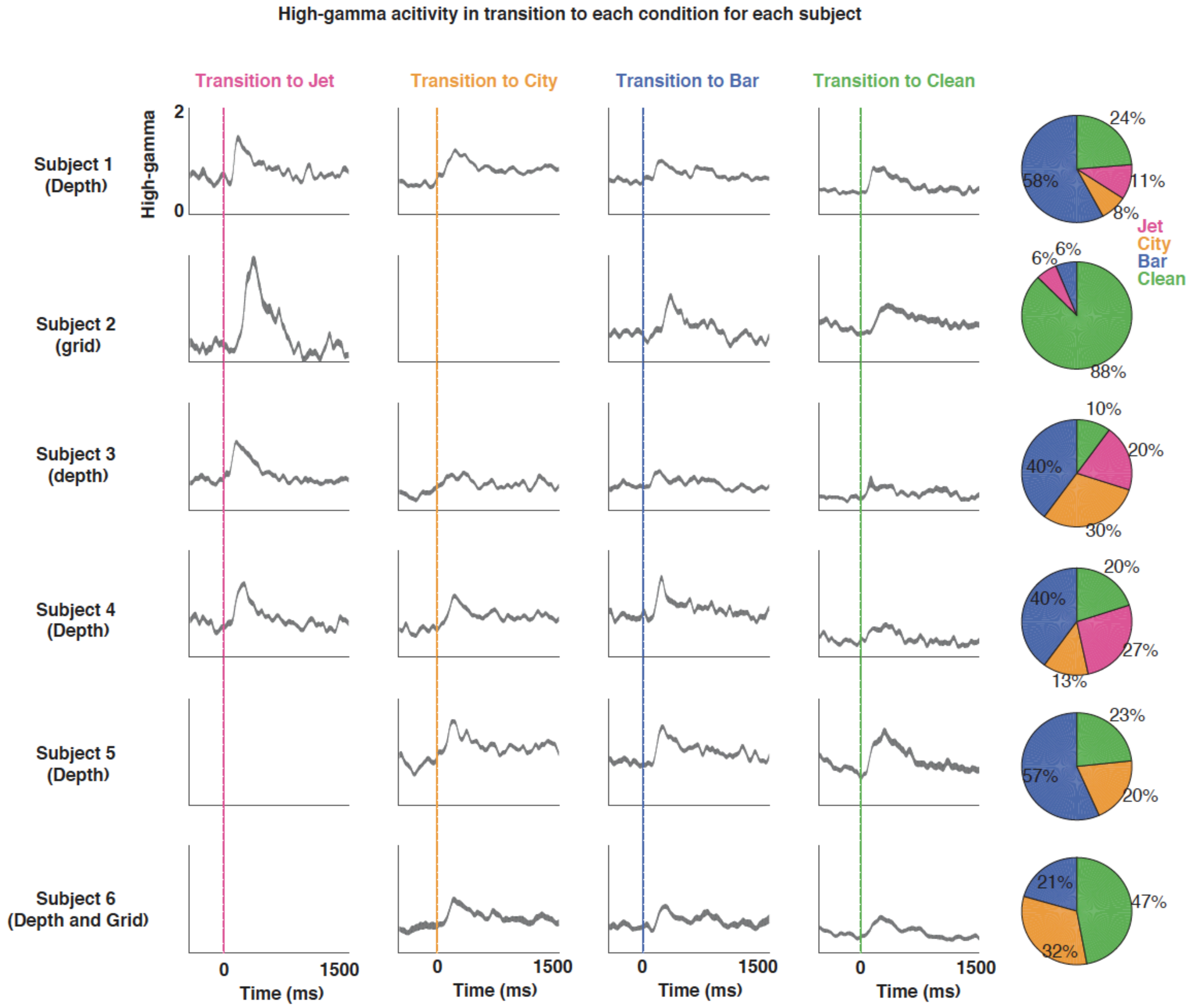

Supplementary Fig. 10. Neural adaptation patterns for individual subjects

The adaptation patterns are plotted for each subject in transition to four background conditions. The plots for each condition show the average response of all electrodes with significant adaptation to that condition. The percentage of electrodes that showed the highest adaptive response to each background condition is shown on the right**.** As an example, in subject 1, 11% of adaptive electrodes are most adaptive to jet, 8% are most adaptive to city, 24% are most adaptive to clean, and 58% are most adaptive to bar noise.

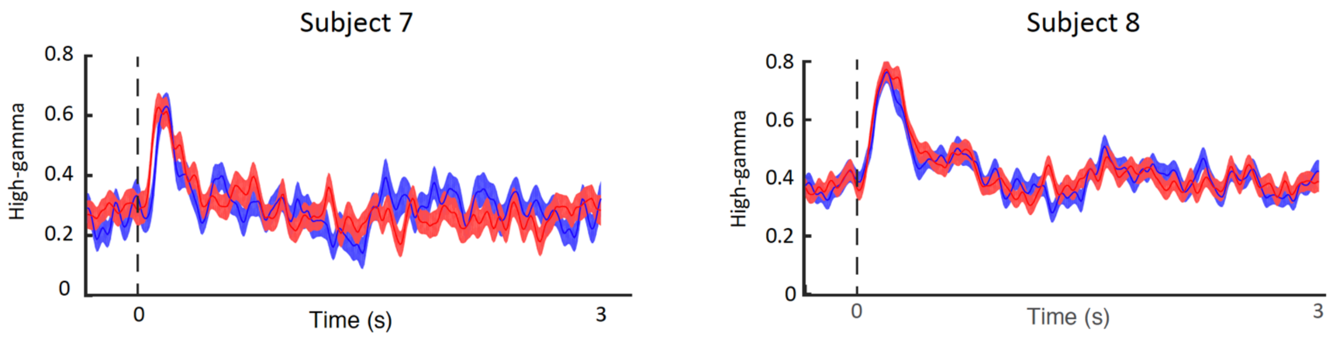

Supplementary Fig. 11. Comparison of neural adaptation in attended and distracted conditions for individual subjects.

The responsive electrodes to speech were selected for subjects 7 and 8. The average of all responsive electrodes in transition to a new noisy condition is shown for both attended (red) and distracted (blue) tasks (Fig. 6). In addition to the average of electrodes, we also did not find any significant difference between the adaptation patterns of attended and distracted at the level of single electrodes.

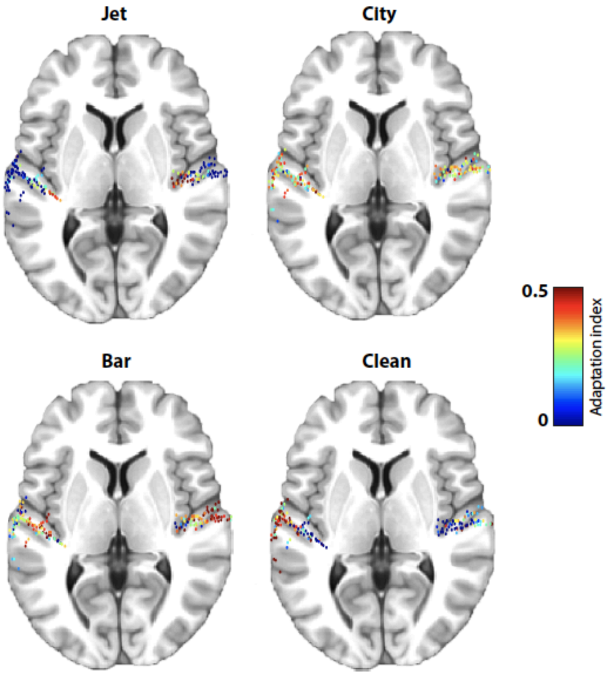

Supplementary Fig. 12. Spatial organization of adaptive responses to each background condition.

The anatomical location of noise indices (AIs) in each background condition is shown on a FreeSurfer template brain, ICBM152.

Correlation with medial-lateral (ML) distance

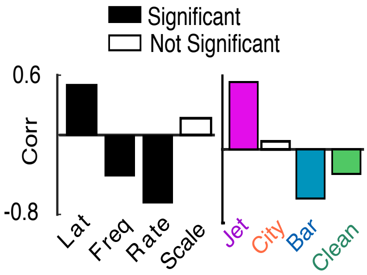

Supplementary Fig. 13. Spatial organization of the tuning parameters.

Left: Correlation between the tuning parameters and medial-lateral distance of electrodes (ML distance). **Right:** Correlation between adaptation indices and ML distance of electrodes (error bar from depth electrodes, n=128)

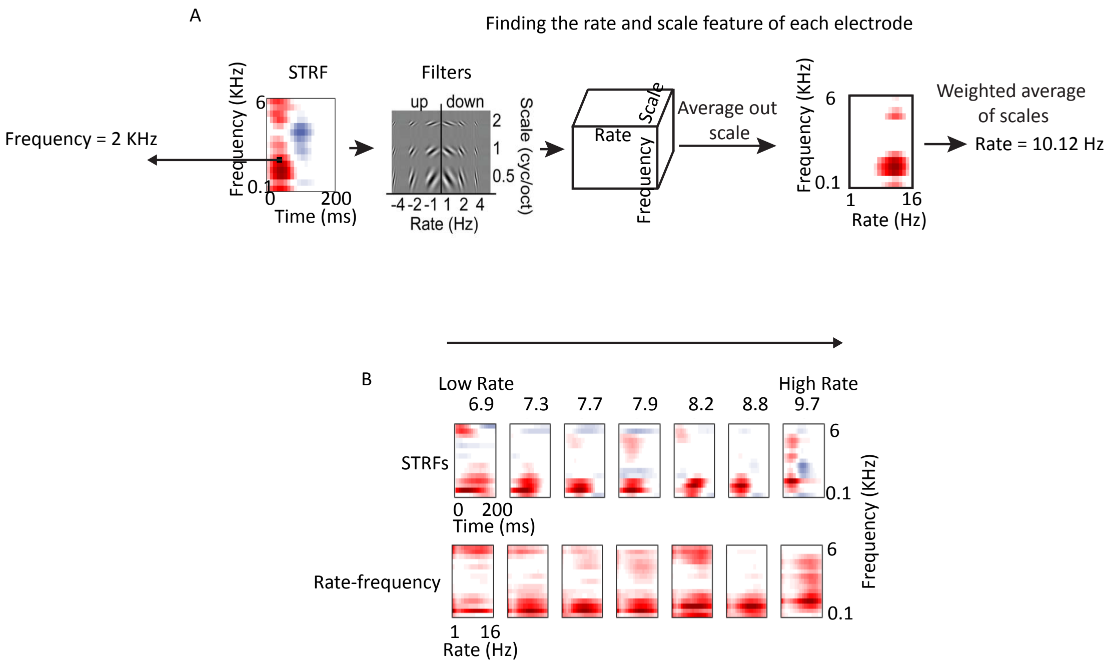

Supplementary Fig. 14. Calculating rate and frequency tuning properties from STRFs of the electrodes.

A) Computations were performed to find the rate-scale model of STRFS. STRFs are passed through a 2D wavelet transform to find the temporal modulation and spectral modulation of the STRF.

B) Example STRFs sorted based on their best rate values. STRFs with higher and lower rates are more sensitive to fast and slow acoustic changes, respectively.
